# Supplementary material for: Chondrocytes in the resting zone of the growth plate are maintained in a Wnt-inhibitory environment
Source: eLife. 2021 Jul 26;10:e64513. doi: 10.7554/eLife.64513 (PMC8313235; doi:10.7554/eLife.64513)
Supplement: Transparent reporting form [file elife-64513-transrepform.docx]

***eLife’s* transparent reporting form**

We encourage authors to provide detailed information *within their submission* to facilitate the interpretation and replication of experiments. Authors can upload supporting documentation to indicate the use of appropriate reporting guidelines for health-related research (see [EQUATOR Network](http://www.equator-network.org/%20)), life science research (see the [BioSharing Information Resource](https://biosharing.org/)), or the [ARRIVE guidelines](http://www.plosbiology.org/article/info:doi/10.1371/journal.pbio.1000412) for reporting work involving animal research. Where applicable, authors should refer to any relevant reporting standards documents in this form.

If you have any questions, please consult our Journal Policies and/or contact us: [editorial@elifesciences.org](mailto:editorial@elifesciences.org).

**Sample-size estimation**

- You should state whether an appropriate sample size was computed when the study was being designed
- You should state the statistical method of sample size computation and any required assumptions
- If no explicit power analysis was used, you should describe how you decided what sample (replicate) size (number) to use

Please outline where this information can be found within the submission (e.g., sections or figure legends), or explain why this information doesn’t apply to your submission:

No statistical method was used to predetermine sample size. We chose the numbers of mice to study based on our prior experience that give good standard errors of the mean and good statistics to make it unlikely that we miss a biologically important difference between groups.

This information can be found within the **Statistical analysis** section of the **Materials and Methods**.

**Replicates**

- You should report how often each experiment was performed
- You should include a definition of biological versus technical replication
- The data obtained should be provided and sufficient information should be provided to indicate the number of independent biological and/or technical replicates
- If you encountered any outliers, you should describe how these were handled
- Criteria for exclusion/inclusion of data should be clearly stated
- High-throughput sequence data should be uploaded before submission, with a private link for reviewers provided (these are available from both GEO and ArrayExpress)

Please outline where this information can be found within the submission (e.g., sections or figure legends), or explain why this information doesn’t apply to your submission:

All experiments were performed in biological replicates. For all data presented in the manuscript, we examined at least three independent biological samples (three different mice) to ensure the reproducibility. Biological replicates were defined as multiple experimental samples sharing common genotypes and genetic backgrounds. For each series of the experiments, all attempts at biological replication were successful. Technical replicates were generated from a single experimental sample. For example, serial sections of the femur growth plate from a single mouse were considered technical replicates. Outliers were uncommon in our datasets and did not impact the trend and the significance of our quantitated results. As a result, all quantitative data were included to ensure transparency in our data interpretation.

This information can be found within the **Replicates** section of the **Materials and Methods**.

The bulk RNA-seq datasets presented herein have been deposited in the National Center for Biotechnology Information (NCBI)’s Gene Expression Omnibus (GEO), and are accessible through GEO Series accession numbers GSE160364 [https://www.ncbi.nlm.nih.gov/geo/query/acc.cgi?acc=GSE160364].

rlog normalized expression counts of the RNA-seq datasets are added as a Supplemental Table (*Col2Q_combined_pairedDE_allrlog.csv*).

This information can be found within the **Data availability** section of the **Materials and Methods**.

The information on the number of independent biological replicates is stated through the **Results** and **Figure Legends**, as well as in a compiled Source Data file included.

**Statistical reporting**

- Statistical analysis methods should be described and justified
- Raw data should be presented in figures whenever informative to do so (typically when N per group is less than 10)
- For each experiment, you should identify the statistical tests used, exact values of N, definitions of center, methods of multiple test correction, and dispersion and precision measures (e.g., mean, median, SD, SEM, confidence intervals; and, for the major substantive results, a measure of effect size (e.g., Pearson's r, Cohen's d)
- Report exact p-values wherever possible alongside the summary statistics and 95% confidence intervals. These should be reported for all key questions and not only when the p-value is less than 0.05.

Please outline where this information can be found within the submission (e.g., sections or figure legends), or explain why this information doesn’t apply to your submission:

We implored a Mann-Whitney’s U-test to carry out independent statistical analyses between our experimental groups. These methods are described in the **Materials and Methods: Statistical analysis** section and data are presented throughout sections of the **Results**. A *p* value of <0.05 was considered statistically significant.

Individual data points were presented wherever statistical tests were used (**Figure 1c; Figure 3b; Figure 4e; Figure 5f; Figure 6e,f,g,h**), along with mean, SD, confidence intervals and exact *p*-values in the **Figure Legends**.

(For large datasets, or papers with a very large number of statistical tests, you may upload a single table file with tests, Ns, etc., with reference to sections in the manuscript.)

**Group allocation**

- Indicate how samples were allocated into experimental groups (in the case of clinical studies, please specify allocation to treatment method); if randomization was used, please also state if restricted randomization was applied
- Indicate if masking was used during group allocation, data collection and/or data analysis

Please outline where this information can be found within the submission (e.g., sections or figure legends), or explain why this information doesn’t apply to your submission:

The experiments were not randomized. We used all the available mice of the desired genotypes. Mice were allocated to particular groups based on results of PCR-genotyping typically performed around one week after birth. Quantitative analyses were conducted using single blind methods by two individuals to ensure unbiased data interpretation.

This information can be found within the **Statistical analysis** and **Imaging and cell quantification** section of the **Materials and Methods**.

**Additional data files (“source data”)**

- We encourage you to upload relevant additional data files, such as numerical data that are represented as a graph in a figure, or as a summary table
- Where provided, these should be in the most useful format, and they can be uploaded as “Source data” files linked to a main figure or table
- Include model definition files including the full list of parameters used
- Include code used for data analysis (e.g., R, MatLab)
- Avoid stating that data files are “available upon request”

Please indicate the figures or tables for which source data files have been provided:

We have provided raw data of quantitative analyses to ensure transparency in the interpretation of our results as a **Source Data** file.
